# Efficacy of Intravitreal rAAV2-ND4 Injection in Treated Versus Fellow Eyes with Leber’s Hereditary Optic Neuropathy: A Meta-Analysis

**DOI:** 10.1080/01658107.2024.2360413

**Published:** 2024-06-10

**Authors:** Amr K. Hassan, Maram Mohsen, Hashem Abu Serhan

**Affiliations:** a Gavin Herbert Eye Institute, University of California, Irvine, California, USA; b Faculty of Medicine, University of Jordan, Amman, Jordan; c Department of Ophthalmology, Hamad Medical Corporation, Doha, Qatar

**Keywords:** Leber’s hereditary optic neuropathy, ND4 injection, intravitreal, rAAV2/2-ND4, lenadogene nolparvovec

## Abstract

To compare the outcomes of rAAV2-ND4 injection in treated versus fellow eyes with Leber’s hereditary optic neuropathy (LHON). The protocol was pre-registered on PROSPERO (CRD42023441669). PubMed, Ovid MEDLINE, Cochrane CENTRAL, Google Scholar, Embase, CrossRef, OpenAlex, and Web of Science were reviewed from 1990–2023. Our analysis included 358 eyes of 307 patients. Of them, 256 (83%) patients received unilateral injections while 51 (17%) received bilateral injections. The mean age was 32 years. Baseline visual acuity (VA) of unilaterally injected eyes was 1.62. At 1 year, it was 1.6 compared to 1.4 (*p* = 0.002) in noninjected eyes. Baseline VA of bilaterally injected eyes was 1.6 and postoperatively at 1.5 years, it became 1.3 (*p* = 0.003). rAAV2/2-ND4 intravitreal injections showed no major differences in terms of improving visual acuity between treated and untreated eyes of the same patient. However, larger prospective RCTs, especially concerning OCT parameters, and visual field, are recommended to provide a better understanding and comparison.

## Introduction

Leber hereditary optic neuropathy (LHON) is a maternally inherited, blinding, bilateral optic neuropathy, currently estimated as the most common mitochondrial disease.^1^ Although the exact prevalence of LHON is unknown, it is estimated to affect 1 in 30,000 to 1 in 50,000 people worldwide.^2^ It primarily affects males, although females can also be affected, albeit to a lesser extent.^3^ The symptoms usually appear in early adulthood, between the ages of 15 and 35, but they can appear at any age.^4^ The most common symptom of LHON is a painless progressive loss of central vision in one eye, which is typically followed by involvement of the fellow eye within weeks or months. The vision loss is usually severe and often results in legal blindness. Typically, peripheral vision remains relatively intact. Other symptoms include dyschromatopsia, large central scotomas, and temporal pallor of the optic disc.^5^

Leber hereditary optic neuropathy is caused by mutations in mitochondrial genes, particularly, m.3460 G>A (MT-ND1), m.11778 G>A (MT-ND4), and m.14484T>C (MT-ND6), with m.11778 G>A being the most frequently reported mutation worldwide.^6^ These mutations affect complex I subunits of the mitochondrial respiratory chain, impairing mitochondrial function and increasing the levels of reactive oxygen species (ROS), resulting in selective loss of retinal ganglion cells (RGCs) and their axons that are particularly sensitive to energy deprivation. This ultimately results in the degeneration of these cells, leading to the characteristic vision loss.^7^

The diagnosis of LHON is typically based on clinical findings, including the characteristic pattern of vision loss and positive family history. Genetic testing can confirm the diagnosis by identifying the specific mitochondrial DNA mutations associated with LHON. If this primary screen is negative and there is a high index of clinical suspicion supported by a maternal mode of inheritance in a patient with a family history, it is recommended to sequence the entire mtDNA to identify one of the rare LHON mutations.^8^

Leber hereditary optic neuropathy may have a strong negative impact on an individual’s quality of life, mainly secondary to the loss of central vision. Affected individuals reported the greatest level of difficulty with reading small print, newspapers, or books. Nevertheless, it’s noteworthy that individuals with LHON can still retain some peripheral vision, which may help in daily activities.^9^ Individuals can cope with the challenges of LHON and maximize their remaining vision by using low-vision aids, visual rehabilitation, and support from healthcare professionals and support groups. Furthermore, genetic counselling is recommended for individuals with LHON and their family members to understand the risk of inheritance better and make informed decisions.^10^

Current treatments for LHON remain inadequate.^11^ However, several experimental treatments have been investigated, including the use of antioxidants, vitamins, and gene therapy, with varying degrees of success. Early intervention, such as the use of idebenone (Raxone, Santhera GmbH), a synthetic antioxidant, may help to improve visual outcomes in some patients,^12^ but failed for others.^13^ The multicopy nature of mtDNA and the current lack of efficient means to directly deliver nucleic acids to the mitochondrial matrix compartment make gene therapy for mitochondrial disorders more challenging.^14^ Over the past decade, substantial progress has been made in the application of gene therapy for inherited retinal diseases. Lenadogene nolparvovec (rAAV2/2-ND4) is a recombinant, replication-defective, adeno-associated virus, serotype 2, containing a modified cDNA encoding the human wild-type mitochondrial ND4 protein and supporting its allotopic expression.^15^ Three ongoing randomized clinical trials (RCTs) are evaluating the efficacy of intravitreal injection of rAAV2/2-ND4 (GS010) in LHON patients with the m.11778 G>A mutation and with vision loss within one year (RESCUE NCT02652767, REVERSE NCT02652780 and REFLECT NCT03293524). REVERSE RCT showed bilateral visual improvement after unilateral intravitreal injection of rAAV2/2-ND4^16^ Some trials investigated unilateral injection,^16–21^ while others evaluated the effect of bilateral rAAV2-ND4 intravitreal injection.^22^ In addition, Yang et al.^23^ evaluated unilateral injection in most of the subjects, except one subject received bilateral injection.

Of note, rAAV2-ND4 comes in several forms; lenadogene nolparvovec (Lumevoq, GenSight Biologics), is a recombinant adeno-associated virus 2 of serotype 2 (rAAV2/2) vector encoding the human wild-type ND4 gene (rAAV2/2-ND4) as well as the rAAV2-ND4 from the Huazhong University of Science and Technology (China).^16–21^ This meta-analysis aims to compare the visual outcomes between treated and nontreated eyes with rAAV2-ND4 intravitreal injection in the treatment of LHON.

## Materials and methods

### Study protocol and database search

This research was carried out following the Preferred Reporting for Systematic Review and Meta-Analysis (PRISMA) recommendations.^24,25^ The study adhered to the tenets of the Declaration of Helsinki and the necessity for institutional review board (IRB) approval was not required since it did not involve human subjects. Our protocol was registered prospectively on PROSPERO [registration number: CRD42023441669]. Meanwhile, on June 2–3, 2023, we searched eight electronic databases [PubMed, Ovid MEDLINE, Cochrane CENTRAL, Google Scholar, Embase, CrossRef, OpenAlex, and Web of Science] to retrieve all studies, from 1990 to 2023, that reported the use of intravitreal rAAV2-ND4 injection in individuals with LHON using the following keywords: ((Leber’s Hereditary Optic Neuropathy) OR (Leber Hereditary Optic Neuropathy) OR (Lebers Hereditary Optic Neuropathy) OR (LHON)) AND ((Treat*) OR (DNA) OR (Gene) OR (Vector) OR (AAV2) OR (ND4)). Medical Subject Headings (MESH) terms were also added whenever applicable to retrieve all relevant studies based on their indexed terms in included databases. Of note, only the first 200 records from Google Scholar were retrieved and screened as per the recent recommendations.^24^ Noteworthy, an updated database search was carried out on just before the data analysis to include any newly published studies before the official synthesis of collected data and yielded no new articles.

Furthermore, after finishing the screening process, we conducted a manual search of references to identify any relevant studies that we could not identify through the original database search. This search was conducted by (1) searching similar articles of the finally included articles in our review through the ‘similar articles’ option on PubMed, (2) searching the reference list of finally included articles in our review, and (3) searching through Google with the keywords used in the original database search.

### Eligibility criteria

We included peer-reviewed original research conducted on human patients suffering from LHON and undergoing treatment with rAAV2-ND4. We included all of the following study designs: randomized clinical trials, cohorts, case-control, case series with more than 5 eyes, and cross-sectional studies. Studies were included regardless of the language of publication. Meanwhile, studies were excluded if they were (1) non-original research (i.e., reviews, commentaries, guidelines, editorials, correspondence, letters to editors, etc.), (2) unavailable full-texts, (3) duplicated records or records with overlapping datasets, (4) case reports and case series with < 5 eyes.

### Screening and study selection

Retrieved records from the database search were exported into EndNote software for duplicate removal before the beginning of the screening phase. Records were then imported into an Excel (Microsoft, USA) spreadsheet for screening. The screening was divided into two steps: title and abstract screening and full-text screening. The full texts of eligible articles were then retrieved for screening before being finally included in the review. Both steps were carried out by two reviewers [AKH, HAS]. Any differences between reviewers were solved through group discussions to reach an agreement.

### Data extraction and assessment of methodological quality and risk of bias

The data extraction was performed by two reviewers [AKH, HAS] through a data extraction sheet that was formatted through Excel (Microsoft, USA). This sheet consisted of fourt parts. The first part included the baseline characteristics of included studies [title, authors’ names, year of publication, country, and study design] and patients as well [sample size, age, and gender]. The second part included data on rAAV2-ND4 (number of injections, laterality, duration of treatment, duration of follow up, preoperative visual acuity, postoperative visual acuity at different time points, optical coherence tomography (OCT) parameters, and visual field (VF) changes). The third part summarized the medical and ophthalmic history of reported cases (i.e., systemic diseases, cardiovascular diseases, cerebrovascular diseases, immunological diseases, history of eye trauma, previous eye diseases, and previous ocular surgeries). The fourth part included the quality assessment of included studies. Methodological quality and risk of bias were assessed using the Cochrane’s revised tool to assess risk of bias in non randomized studies (ROBINS-I). In addition, author-reported conflicts of interest and industry sponsorships were collected for all studies.

For the patient demographics, age was extracted according to the age at the time of treatment onset and not at the time of disease onset. Timing of intervention was calculated as the duration between the age of onset and the age of treatment, if provided, and if not provided, it was extracted from the duration of vision loss. The duration of follow-up was provided by the studies as the duration of follow-up visits after the administration of intravitreal injections.

### Data synthesis

Acquired data was tabulated and reorganized, then qualitative and quantitative analysis were performed. Qualitative analysis was done through table columns comparison using the Statistical Package for Social Sciences (SPSS) version 27 (IBM SPSS Corp, SPSS Statistics ver. 26, USA). Descriptive analysis was used to display categorical variables as percentages and frequencies while presenting numerical variables as a mean and standard deviation to evaluate the data quantitatively. The significance of the data was determined using a categorical Chi-square test. All statistical tests were conducted with a 95% confidence interval and a 5% error margin. A p-value of less than 0.05 was considered statistically significant. Quantitative analysis was performed on categorical basis through meta-analysis executed using Cochrane’s Review Manager (RevMan), version 5.4 (The Cochrane Collaboration’s guidelines, 2020). The random effect model is used when heterogeneity between studies is present, and the fixed effect model is used when heterogeneity is absent. Images and graphs were analysed using PlotDigitizer.

## Results

**Figure 1.**
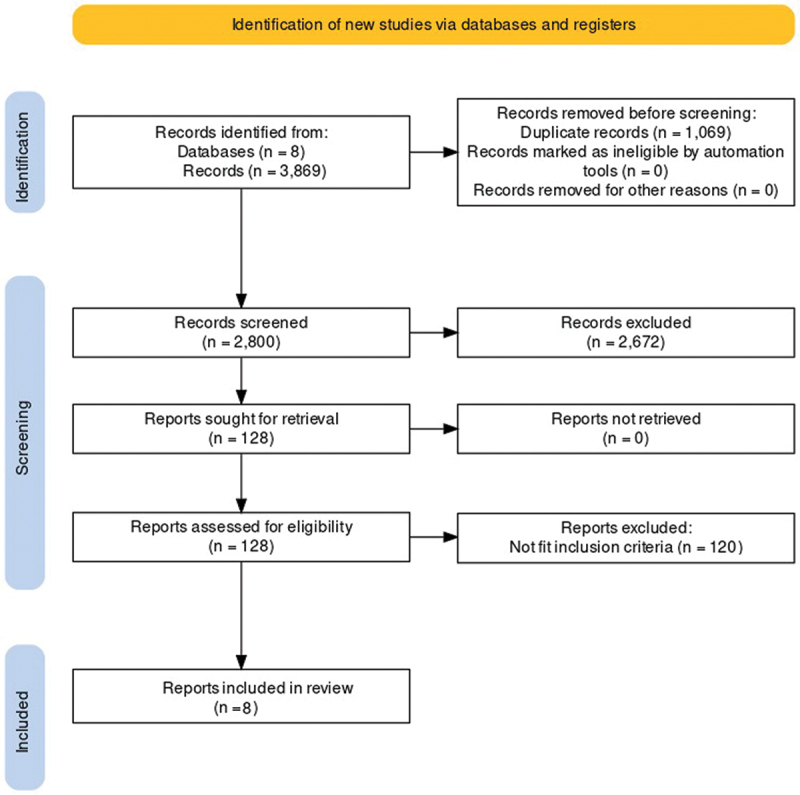
PRISMA figure for the literature search process for the meta-analysis.

Our literature review disclosed 3869 peer-reviewed publications. After title and abstract screening and removing duplicate publications (1069 duplicates removed), we excluded 2672 publications. After reading the full manuscript of the remaining 128 articles and applying the inclusion and exclusion criteria, we only included 8 articles. (Figure 1) All of the studies were prospective case controls. None of the studies had a high risk of bias when applying Cochrane’s revised tool to assess the risk of bias non in randomized studies (ROBINS I). Only one study in our review directly compared treated to untreated eyes.^19^

### Baseline characteristics

Our analysis included 358 eyes of 307 patients. Of them, 256 (83%) patients received unilateral injections while 51 (17%) received bilateral injections. The entire group consisted of 235 (77%) males and 72 (23%) females. The mean age of the patients was 32 years (standard deviation ±15, range = 9–74). All of the patients (100%) had mt11778G>A mutation. All patients received a single intravitreal injection of rAVV2-ND4 at a mean dose of 7.9 × 10 vg in 90 microlitres per eye. They were followed up for a mean of 28.7 months. Tables 1 and 2 show the baseline characteristics of the included studies.

**Table 1.** Baseline characteristics of included studies.

Character		Number of Studies	Studies
Year	2016	2	Yang et al., 2016; Wan et al., 2016
	2020	1	Yu-Wai-Man et al., 2020
	2021	4	Vignal-Clermont et al., 2021; Biousse et al., 2021; Li et al., 2021; Newman et al., 2021
	2023	1	Newman et al., 2023
Country	China	3	Yang et al., 2016; Wan et al., 2016; Li et al., 2021
	UK	1	Yu-Wai-Man et al., 2020
	France	1	Vignal-Clermont et al., 2021
	USA	3	Newman et al., 2023; Biousse et al., 2021; Newman et al., 2021
Type of Studies	Prospective	8	Yu-Wai-Man et al., 2020; Vignal-Clermont et al., 2021; Newman et al., 2023; Biousse et al., 2021; Yang et al., 2016; Newman et al., 2021; Wan et al., 2016; Li et al., 2021

**Table 2.** Subject characteristics in each study.

Study	Number of Subjects	Mean Age in Years (SD, Range)	Male:Female	Timing of Intervention (Interval between Diagnosis and Treatment in Months) Mean (Range)	Baseline Mean VA treated eye (SD)	Baseline VA fellow eye (SD)	Mean Follow Up Duration in Weeks
Yu-Wai-Man et al., 2020 (REVERSE)	37	34.2 (15.2, 15–67)	29:8	8.8 (6–12)	1.67 (0.5)	1.55 (0.42)	96
Vignal-Clermont et al., 2021 (REVEAL)	15	47.9 (17.2)	2:13	70.8 (7.2–266.4)	2.29 (0.72)	2.03 (0.68)	257
Newman et al., 2023	98 (148 treated eyes)	32.1 (13.8, 15–74)	78:20	8.3 (1.7–11.9)	1.59 (0.47)	1.5 (0.45)	78.2
Biousse et al., 2021 (RESTORE)	61	35.1 (15.4, 15–69)	48:13	7.4 (2.3–12.8)	1.62 (0.55)	1.62 (0.55)	257.1
Yang et al., 2016	9 (10 treated eyes)	19.2 (10.9)	7:2	NA	1.61 (0.4)	1.36 (0.5)	321.4
Newman et al., 2021 (RESCUE)	38	36.8 (15.4, 15–69)	31:7	3.6 (1.3–6)	1.31 (0.52)	1.26 (0.62)	96
Wan et al., 2016	9	19.2 (11.6, 9–46)	7:2	10.8 (0–24)	1.69 (0.43)	1.4 (0.54)	38.6
Li et al., 2021	40	20.3 (6.4, 12–42)	33:7	41.3 (7–312)	1.83 (0.47)	1.75 (0.46)	52.1

### Visual acuity

**Figure 2.**
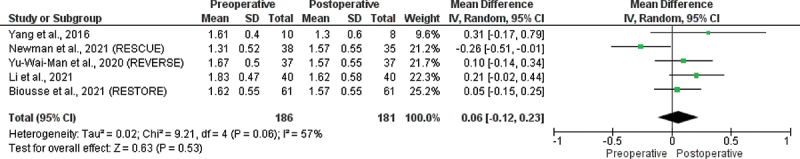
Preoperative and postoperative VA changes in the unilaterally injected eyes at one year.

For the unilateral injected eyes, baseline visual acuity was 1.62 (±0.5, Range = 1.3–2.5, 307 patients). At 3 months, it was 1.4 (±0.5, Range = 1.4–1.4, 17 patients, 2 studies,^21,23^ *p* = 0.078). At 6 months it was 1.4 (±0.5, Range = 1.2–1.5, 78 patients, 3 studies,^17,21,23^ *p* < 0.001) At 1 year, it was 1.6 (±0.6, Range = 1.3–1.62, 181 patients, 5 studies,^16–19,23^ *p* = 0.692). At 2 years, it was 1.4 (±0.6, Range = 1.3–1.5, 141 patients, 4 studies,^16,17,19,23^ *p* < 0.001). Figure 2 shows preoperative and postoperative VA changes in the unilateral injected eyes at one year.

**Figure 3.**
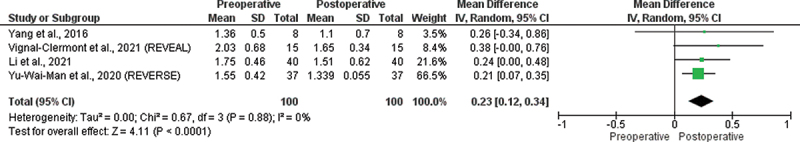
Preoperative and postoperative VA changes in the noninjected eyes at one year.

For the non injected eyes, baseline visual acuity was 1.6 (±0.5, Range = 1.3–2, 179 patients^16,18,20,22,23^), and at 1 year postoperatively, it was 1.4 (±0.5, Range = 1.1–1.7, 100 eyes,^16,18,20,23^ *p* = 0.002). At 1.5 years, it was 1.4 (±0.5, Range = 1.2–1.5, 80 eyes,^16,19,23^ *p* = 0.003). Figure 3 shows preoperative and postoperative VA changes in the noninjected eyes at one year.

For bilaterally injected eyes, the preoperative mean visual acuity for the first eye was 1.6 (±0.5, 50 eyes^22^) and for the second eye was 1.4 (±0.5, 50 eyes^22^) and postoperatively at 1.5 years, it became 1.3 (±0.5, 50 eyes,^22^ *p* = 0.003) for the first eye was and 1.4 (±0.6, 50 eyes,^22^ *p* = 0.1) for the second eye.

### OCT parameters

For the injected eyes, the preoperative Retinal Nerve Fiber Layer (RNFL) papillomacular bundle mean thickness was 28.7 microns (±10.2, Range = 23.1–34.1, 75 patients^16,19^), postoperatively at 2 years, it became 23.6 (±1.3, Range = 22.9–24.3, 75 patients,^16,19^ *p* < 0.001). The temporal RNFL was reported to be at a mean thickness of 38.6 microns (±18, Range = 27.5–49.5, 75 patients^16,19^), and postoperatively at 2 years, it became 35.6 (±9.9, Range = 25.7–45.3, 75 patients,^16,19^ *p* = 0.208). Retinal Ganglion Cell (RGC) Layer macular volume was 0.6 mm^3^ (±0.2, Range = 0.53–0.74, 75 patients^16,19^), and at 2 years, it became 0.52 mm^3^ (±0.016, Range = 0.52:0.53, 75 patients,^16,19^ *p* < 0.001).

For the non injected eyes, the preoperative RNFL Papillomacular bundle mean thickness was 29.7 (±15.9, Range = 23.6–35.7, 75 patients^16,19^), postoperatively at 2 years it became 23.3 (±1.5, Range = 22.4–24.3, 75 patients,^16,19^ *p* < 0.001). The preoperative temporal RNFL mean thickness was 39.7 (±20.7, Range = 28.9–50.3, 75 patients^16,19^), and at 2 years, it became 25.5 (±1.6, Range = 24.2–26.9, 75 patients,^16,19^ *p* < 0.001). The preoperative RGC macular volume was 0.6 (±0.2, Range = 0.5–0.7, 75 patients^16,19^), and at 2 years, it became 0.5 mm^3^ (±0.015, Range = 0.5–0.51, 75 patients,^16,19^ *p* < 0.001).

### Visual field

The injected eye group baseline visual field mean deviation was −22.5 (±10.3, Range = −29.4 – −16.3, 93 patients^16,19^). Two years postoperatively, it was −23.5 (±1.3, Range = −23.3 – −23.6, 75 patients,^16,19^ *p* = 0.429)

The non injected eye group baseline visual field mean deviation was −21.5 (±11.1, Range = −28.7 – −16.7, 83 patients^16,19^). Two years postoperatively, it was −22.9 (±1.37, Range = −22.4 – −23.5, 75 patients,^16,19^ *p* = 0.266). Table 3 shows preoperative and postoperative changes following rAAV2-ND4 intravitreal injection.

**Table 3.** Preoperative vs Postoperative changes following rAAV2-ND4 intravitreal injection.

	Injected Eyes			Non-Injected Eyes		
	Preoperative	Postoperative	*p* value	Preoperative	Postoperative	*p* value
Visual Acuity (LogMAR)	1.62 (0.5)	1.6 (0.6)*	0.692	1.6 (0.5)	1.4 (0.5)*	**0.002**
RNFL PMB Mean Thickness (Microns)	28.7 (10.2)	23.6 (1.3)**	**<0.001**	29.7 (15.9)	23.3 (1.5)**	**<0.001**
Temporal RNFL Thickness (Microns)	38.6 (18)	35.6 (9.9)**	0.208	39.7 (20.7)	25.5 (1.6)**	**<0.001**
RGC Macular Volume (mm^3^)	0.6 (0.2)	0.52 (0.016)**	**<0.001**	0.6 (0.2)	0.5 (0.015)**	**<0.001**
Visual Field Mean Deviation (dB)	−22.5 (10.3)	−23.45 (1.3)**	0.429	−21.5 (11.1)	−22.9 (1.4)**	0.266

*At 1 year postoperatively.

**At 2 years postoperatively.

*p* values are for the difference between the pre intervention and the post intervention stages

LogMAR: Logarithm of Minimal Angle of Resolution.

RNFL: Retinal Nerve Fiber Layer.

PMB: Papillomacular Bundle.

RGC: Retinal Ganglion Cell.

### Complications

There were 163 (44%) incidences of intraocular inflammation, however, 109 (67%) of them were in one study, in which patients received bilateral injections.^22^ Other complications included 27 episodes (7%) of anterior uveitis, 25 episodes (7%) of intermediate uveitis, both also only reported in one study,^16^ as well as 27 episodes (7%) of elevated intraocular pressure,^22^ and 21 episodes (6%) of vitritis,^20^ which were both also reported in a single study, respectively.^20^ This unilateral and uneven reporting of complications, in the absence of major differences in dosage or technique, suggests a possible over-reporting bias or an operator issue.

## Discussion

Leber’s hereditary optic neuropathy results from a point mutation in mtDNA, most commonly m.11778 G>A, which inhibits NADH dehydrogenase activity in complex I of the mitochondrial electron transport chain, leading to a decrease in energy production by the mitochondria, and therefore optic nerve cells atrophy can occur.^26^ Since the mitochondrial inheritance pattern of LHON, it has a strong male preponderance (80% to 90%), and the usual age at onset is between 15 to 35 years. This correlates with our analysis where 235 (77%) males and 72 (23%) females were reported. In addition, the mean age of the patients was 32 years (standard deviation ±15, range = 19.2–47.9).^2^ The most common reported mutation worldwide is m.11778 G>A with having also the worst prognosis among other mutations.^27^ All of the included subjects (100%) had mt11778G>A mutation hence they were treated with Lenadogene nolparvovec.

It has been hypothesized that rAAV2/2-ND4 injections increase energy supply to the optic nerve cells by supplying normal ND4 to the mitochondria, allowing for the restoration of optic function.^28^ Unilateral rAAV2/2-ND4 injected eyes showed worse visual acuity results at one year (1.6) compared to noninjected eyes (1.4). However, these results were not the case after 2 years of follow-up, where almost similar visual function was found. This could be attributed to the progressive nature of the disease.^29^ Bilateral injected eyes showed stable or even improved visual acuity results at 1.5 years.

To accurately assess the therapeutic efficacy of any treatment, the definition of visual recovery should be standardized between studies. Yang et al.^30^ defined visual recovery as an improvement of at least 0.3 logMAR from baseline. Lam et al.^31^ defined it as improvement from a baseline of at least 15 ETDRS letters. Klopstock^32^ used definition criteria as the ability to read ≥ 10 additional ETDRS letters (equivalent to 20.2 logMAR). These varied definitions of ‘recovery’ from study to study make the comparison between them challenging. An international consensus to set definition criteria of visual recovery is highly recommended to formulate a complete idea about the long-term results of the treatment.

Our analysis revealed improvement in OCT parameters including RNFL papillomacular bundle thickness, temporal RNFL thickness, and RGC macular volume in all injected eyes compared to noninjected eyes at all follow-up points. Retaining RNFL and RGC functions is fundamental to keeping the optic nerve functional. Wan et al.^21^ found loss of vision in LHON patients was related to the condition of the optic nerve and RNFL status not to the duration of the disease.

The bilateral effect of unilateral injections of Raav2/2-ND4 is particularly important since studies often note a contralateral improvement of visual acuity and the search for an explanation for this phenomenon is an important direction of research. This contralateral improvement was highlighted by the observation of the presence of viral vector DNA in not only the injected eye but also in tissues of the uninjected eye in cynomolgus monkeys.^19^ Since rAAV2/2-ND4 DNA was detected in the optic chiasm of injected animals, the anatomic route taken by the viral vector DNA to transfer from the treated eye to the nontreated eye was hypothesized to be via the optic nerve and chiasm (through anterograde and subsequent retrograde transport along the optic projections). Still, a systemic transfer of rAAV2/2-ND4 DNA, which would require biodissemination studies for the presence of rAAV2/2-ND4 DNA in blood, as well as transfer of mitochondrial material (eg, mRNA or proteins), both cannot be excluded at this stage.^33^ Finally, brain plasticity could partially account for the improvement of visual function in the contralateral eye, as reported in some blind subjects implanted with a retinal prosthesis.^34^

Regarding OCT parameters, both injected and non-injected eyes demonstrated a reduction in the RNFL papillomacular bundle mean thickness, the temporal RNFL mean thickness, as well as the RCG macular volume. Furthermore, the visual field mean deviation has worsened in both treated and untreated eye groups. Despite the low number of studies reporting on these outcomes, apart from the visual field mean deviation change, most of the other outcomes were statistically significant as seen in Table 2.^16,19^ These changes, being similar in magnitude between both treated and untreated, except for the decrease in temporal RNFL thickness which was larger in magnitude in the untreated eyes vs the treated eyes, suggest that these changes are due to irreversible changes in the disease process, which are not affected by treatment, and warrant a more in-depth look into these outcomes.

However, the current findings are inconclusive and need further investigation due to several limitations. First, most studies evaluated unilateral intravitreal rAAV2/2-ND4 injections, with only one study assessing bilateral injections.^22^ Therefore, the majority of our included subjects received unilateral injections (83%) compared to only 17% who received bilateral injections. Furthermore, only one of the studies directly compared the eyes to each other, so we were obligated to perform an indirect meta-analysis.^19^ We recommend large prospective RCTs investigating the laterality effect of rAAV2/2-ND4 injections on the long-term prognosis. Furthermore, this analysis doesn’t take an account the quality of life (QoL) improvement in the follow-up period after unilateral compared to bilateral injections. Future studies assessing the QoL of subjects with LHON are warranted.

## Conclusion

rAAV2/2-ND4 intravitreal injections showed no major differences in terms of improving visual acuity between treated and untreated eyes of the same patient. However, larger prospective RCTs, especially concerning OCT parameters, and visual field, are recommended to provide a better understanding and comparison.

## Data Availability

The datasets used and/or analysed during the current study are available from the corresponding author on reasonable request.
